# Diagnostic and prognostic value of circulating miRNA‐499 and miRNA‐22 in acute myocardial infarction

**DOI:** 10.1002/jcla.23332

**Published:** 2020-06-11

**Authors:** Xiaoqing Wang, Lu Tian, Qiyu Sun

**Affiliations:** ^1^ Clinical Laboratory Hospital Affiliated to Chengde Medical University Chengde China

**Keywords:** biomarkers, miRNA‐22, miRNA‐499, myocardial infarction

## Abstract

**Background:**

Currently, acute myocardial infarction (AMI) represents a serious cardiovascular disease with high morbidity and mortality. Therefore, this study aimed to systematically evaluate the roles of miRNA‐499 and miRNA‐22 as potential biomarkers for AMI.

**Methods:**

According to the inclusion and exclusion criteria, we measured circulating levels of miRNAs in 50 AMI patients and 50 non‐MI populations. The expression levels of plasma miRNA‐499 and miRNA‐22 were analyzed by real‐time fluorescent quantitative polymerase chain reaction (qRT‐PCR). A statistical analysis of clinical data of AMI patients was conducted by 90‐day follow‐up.

**Results:**

Real‐time PCR analysis showed that the relative expression level of miRNA‐499 increased gradually among the three groups (*P* < .05). However, the expression of miRNA‐22 showed a downward trend (*P* < .05). According to logistic analysis, the relative levels of miRNA‐499 and miRNA‐22 were important predictors of AMI. When the miRNA‐499 and miRNA‐22 levels were 0.377 and 0.946 separately, the diagnostic value of miRNA‐499 and miRNA‐22 for AMI was 86.00% and 86.00% for sensitivity, and 98.00% and 94.00% for specificity, respectively. In addition, compared to the baseline GRACE scoring system, the combination of miRNA‐499, miRNA‐22, and GRACE scores had a stronger discriminating power for MACE occurrence, with a sensitivity of 100.00% and a specificity of 79.40%.

**Conclusions:**

The results showed that plasma miRNA‐499 and miRNA‐22 were more sensitive and specific for the diagnosis of AMI, suggesting that they can be used as potential biomarkers for clinical diagnosis of AMI.

## 1 INTRODUCTION

At present, coronary heart disease (CHD) has been the main cause of mortality in both developed and developing countries, with acute myocardial infarction (AMI) accounting for the majority.^1^ Due to the high morbidity and high mortality of AMI, early diagnosis and timely treatment are particularly important.^2^ Currently, creatine kinase isoenzyme (CK‐MB) and troponin (T or I) are considered as important indicators of AMI^3^ and have been widely used in clinical practice.^4^ However, other diseases, such as congestive heart failure, myocarditis, renal failure, and skeletal muscle tissue damage, also lead to elevated serum levels, which reduce the diagnostic specificity.^5^ Consequently, it is especially important to explore and research new biomarkers in the diagnosis of AMI.^6^ GRACE risk score is the current risk assessment tool recommended for acute coronary syndromes. It can effectively assess the risk of death or AMI in hospitalization or discharge for 6 months.^7^, ^8^ Recently, some studies have shown that combining new biomarkers with GRACE risk scores can improve cardiovascular risk prediction ability compared to biochemical indicators alone.^9^

MicroRNAs (miRNAs) are a class of noncoding, small single‐stranded RNAs.^10^ Mature miRNAs can bind to the 3' untranslated region of the target gene mRNA^11^ and ultimately inhibit gene expression by promoting mRNA degradation or inhibiting translation.^12^ Many studies have shown that miRNAs are involved in a variety of gene regulation processes such as proliferation, development, differentiation, inflammation, and other physiological processes.^13^, ^14^ MiRNAs are considered to be biomarkers of certain diseases such as cardiovascular diseases because they are stable in blood and other body fluids.^15^, ^16^ Similarly, Wang et al^17^ also believed that serum miRNAs may be promising as novel indicators for the diagnosis of AMI. In addition, many studies have shown that circulating miRNAs play an important role in the poor prognosis of patients with AMI.^18^ Recently, miRNA‐499 has been shown to have a crucial effect on the differentiation of cardiac stem cells into cardiomyocytes. And the literature indicated that the concentration of miRNA‐499 was significantly elevated in patients with myocardial infarction.^19^ Furthermore, Huang et al^20^ found that miRNA‐22 in the heart of high expression was an important regulatory factor of cardiac remodeling, highlighting miRNA‐22 as a candidate biomarker for cardiovascular disease. However, other research reports on the diagnostic performance of serum miRNA‐499 on AMI are still controversial, and the diagnostic value of miRNA‐22 in AMI is rarely reported.

Therefore, the main purpose of this study was to explore the diagnostic value of miRNA‐499 and miRNA‐22 in AMI. Furthermore, given the prognostic significance of GRACE score, we aim to identify the potential predictive value of miRNA‐499 and miRNA‐22 on the GRACE score to guide risk stratification and clinical treatment.

## 2 METHODS

### 2.1 Participants

The study included 50 AMI patients (AMI), 25 patients with unstable angina (UAP) and 25 healthy subjects (HC) who were admitted to the Affiliated Hospital of Chengde Medical College from October 2018 to July 2019.

The AMI diagnostic criteria were based on the 2018 ESC/ACCF/AHA/WHF Fourth universal definition of myocardial infarction.^5^ The unstable angina was diagnosed with reference to the criteria recommended by Chacko KA.^21^ The healthy group was a healthy subject who had not been diagnosed with cardiovascular disease.

Patients will be excluded if they have the following medical history: the other history of heart disease, severe infection, severe liver and kidney dysfunction, malignant tumors, autoimmune diseases, etc

The design of this study was complied with the Declaration of Helsinki. This research protocol was approved by the Ethics Committee of the Affiliated Hospital of Chengde Medical College. No approval number is applicable.

### 2.2 Sample collection and storage

The venous blood of the patients after 6 hours of admission was collected using an EDTA‐anticoagulative tube of about 5 mL. After centrifugation at 3000 *g* for 10 minutes, the supernatant was transferred to an RNase/DNase‐free tube and stored at −80°C for analysis.

### 2.3 Total RNA extraction

The total RNA was isolated from plasma using TRIzol LS Reagent (Invitrogen) according to the manufacturer's instructions. The synthesized miRNA cel‐miR‐39 (RiboBio Co) was added as an internal control to each sample to be extracted at a final concentration of 10^−4^ pmol/L. Finally, the RNA pellet was dissolved in 10μl of DEPC water and stored at −80°C. The Biospec‐mini UV‐Visible spectrophotometer was applied to verify the quality of total RNA.

### 2.4 Quantitative reverse transcription polymerase chain reaction (qRT‐PCR)

The reverse transcription reaction was carried out using the Bulge‐LoopTM miRNA qRT‐PCR primer set (RiboBio Co). Caenorhabditis elegans microRNA (cel‐miR‐39) was used as the control. The reverse transcription process was carried out at 42°C for 60 minutes and then at 70°C for 10 minutes. At last, the resulting cDNA was stored at −20°C until use.

The PCR was carried out according to the Bulge‐LoopTM miRNA qRT‐PCR Kit (RiboBio Co). The final reaction volume was 20 μL of reaction and was performed on a Roche Cobas z480 detection system (Roche Molecular Diagnostics). Amplification reaction program: After initial denaturation for 2 minutes at 95°C, and then 40 cycles at 95°C for 15 seconds, 60°C 30 seconds and 95°C 15 seconds. Finally, a melting curve is produced. The data were obtained directly from a real‐time fluorescent quantitative PCR instrument using an amplification profile of cel‐miR‐39 as an internal standard. To calculate the relative expression levels of miRNAs, the 2^−ΔCt^（ΔCt = Ct_miRNAs_−Ct_cel‐miR‐39_）method was used to assess miRNA expression.^22^, ^23^

### 2.5 Follow‐up and study end point

A 90‐day follow‐up was performed on patients with AMI and UAP by telephone or hospitalization. The study end point was the occurrence of cardiovascular adverse events, including MACE, all‐cause death, myocardial infarction, cardiogenic shock, and cardiac arrest/ventricular fibrillation.

### 2.6 Calculation of GRACE risk score

GRACE scores were based on age, heart rate, systolic blood pressure, creatinine, Killip classification, prehospital cardiac arrest, ST‐segment depression, and elevated myocardial enzymes in all patients. The scoring criteria were based on the GRACE risk score.^24^

### 2.7 Statistical analyses

Statistical analysis was performed using SPSS 25.0 software (SPSS). All data are expressed as mean ± standard error (SE). All continuous variables were checked using the Kolmogorov‐Smirnov normality test to show their distributions. One‐way ANOVA was used to test for differences among the three groups, and independent‐samples *t* test was used between the two groups. Binary logistic regression analysis was performed to show the associated variables in the data analysis to determine the independent predictors of AMI. Receiver operating characteristic curves further analyzed the diagnostic efficacy of the two indicators for AMI. Correlation between variables in AMI patients was analyzed by Spearman rank correlation. All statistical tests were two‐tailed, and *P* < .05 was considered statistically significant.

## 3 RESULTS

### 3.1 Clinical characteristics of the study population

In this study, 50 patients with AMI served as the experimental group (AMI). And 25 UAP patients (UAP) and 25 healthy people (HC) served as the control. As shown in Table 1, there were significant differences in white blood cell counts, red blood cell counts, and total cholesterol levels and triglycerides among the groups (*P* < .05).

**Table 1:** Baseline characteristics of all patients

Variables	HC group (n = 19)	UP group (n = 20)	MI group (n = 40)	*P*
Age (years)	57.48 ± 12.89	61.29 ± 10.27	59.20 ± 12.50	.409
Gender, male	13	15	37	.141
WBC (10^9^/L)	6.02 ± 1.05	6.09 ± 1.51	10.18 ± 4.17	.000
RBC (10^12^/L)	4.96 ± 0.53	4.45 ± 0.41	4.75 ± 0.50	.002
Hb (g/L)	147.44 ± 12.64	148.72 ± 65.20	146.40 ± 16.57	.964
PLT (10^9^/L)	224.72 ± 51.39	238.50 ± 70.70	222.54 ± 59.64	.422
TC (mmol/L)	4.54 ± 0.77	3.87 ± 1.30	4.76 ± 1.23	.008
TG (mmol/L)	0.97 ± 0.29	1.82 ± 1.28	1.58 ± 0.83	.002
BUN (mmol/L)	5.44 ± 1.14	5.49 ± 2.94	6.07 ± 1.20	.250
Cr (µmol/L)	69.52 ± 14.50	80.55 ± 79.27	78.06 ± 51.77	.745

Abbreviations: BUN, urea nitrogen; Cr, creatinine; Hb, hemoglobin; PLT, platelet count; RBC, red blood count; TC: total cholesterol; TG: triglyceride; WBC, white blood cell count.

As shown in Table 2, 50 patients with myocardial infarction (AMI) and 50 patients with non‐myocardial infarction (non‐MI) (UAP + HC) were compared. White blood cell count was significantly higher in the AMI group compared with the non‐MI group (*P* < .05).

**Table 2:** Analysis of the AMI group and non‐MI group (non‐MI)

Variables	Non‐MI	AMI	*P*
Age (years)	59.76 ± 11.96	59.20 ± 12.50	.508
Gender, male	28	37	.07
WBC (10^9^/L)	6.09 ± 1.29	10.18 ± 4.17	.000
RBC (10^12^/L)	4.71 ± 0.53	4.75 ± 0.50	.772
Hb (g/L)	148.08 ± 46.48	146.40 ± 16.57	.423
PLT (10^9^/L)	233.20 ± 61.94	222.54 ± 59.64	.833
TC (mmol/L)	4.20 ± 1.09	4.76 ± 1.23	.654
TG (mmol/L)	1.39 ± 0.99	1.58 ± 0.83	.741
BUN (mmol/L)	5.47 ± 2.21	6.07 ± 1.20	.291
Cr (µmol/L)	75.03 ± 56.67	78.06 ± 51.77	.836
miRNA‐499	0.09 ± 0.12	1.93 ± 1.49	.000
miRNA‐22	2.04 ± 1.50	0.44 ± 0.36	.000

### 3.2 Comparison of serum miRNA‐499 and miRNA‐22 levels

**FIGURE 1:**
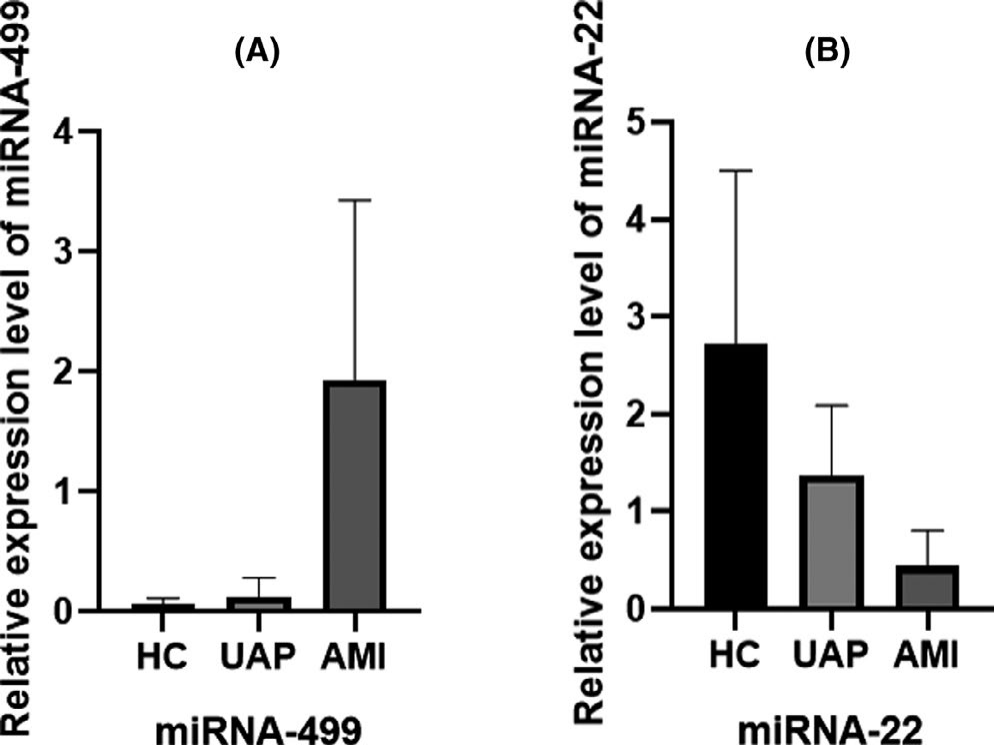
Comparison of serum miRNA‐499 and miRNA‐22 among three groups

As a result, the mean levels of miRNA‐499 in the HC, UAP, and AMI groups were 0.05 ± 0.05, 0.12 ± 0.15, and 1.93 ± 1.49, respectively. As shown in Figure 1A, the serum miRNA‐499 levels were significantly up‐regulated in AMI patients compared to subjects in other groups including healthy controls and UAP patients. As shown in Figure 1B, the mean concentrations of miRNA‐22 among HC, UAP, and AMI were 2.71 ± 1.78, 1.37 ± 0.72, and 0.44 ± 0.36, respectively. Contrary to the expression trend of miRNA‐499, the level of miRNA‐22 was gradually reduced among the three groups. In the multiple comparisons among the groups, significant statistical differences of miRNA‐22 were found (*P* < .05).

**FIGURE 2:**
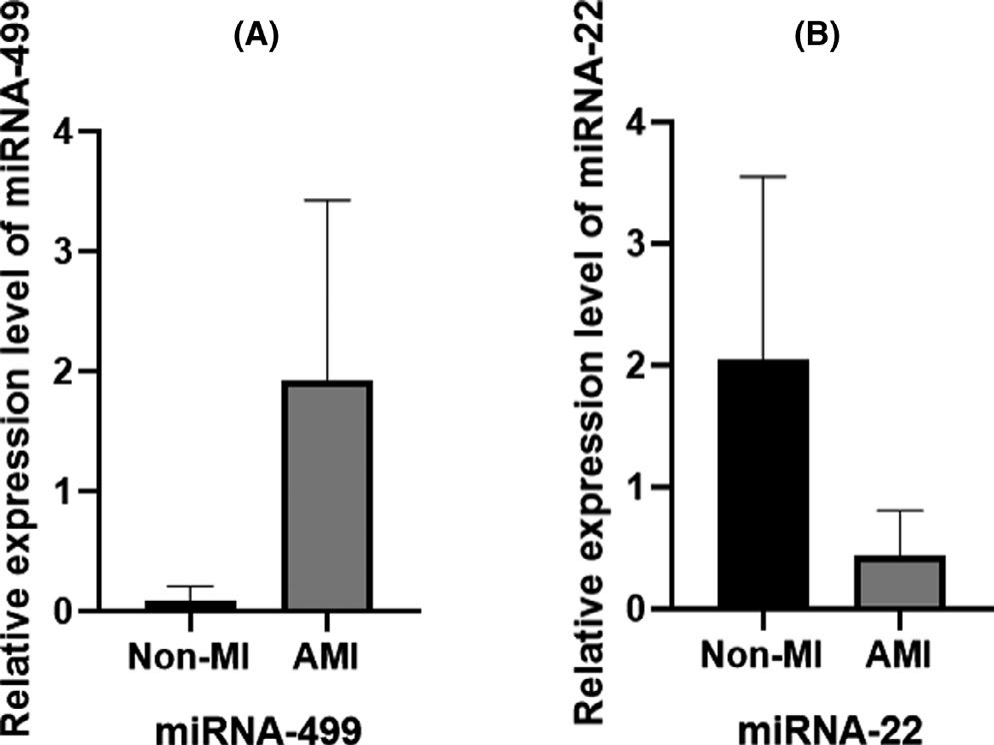
Comparison of miRNA‐499 and miRNA‐22 between non‐MI group and AMI group

Among these patients, it was observed that the relative expression levels of miRNA‐499 were significantly higher in the AMI group than in the non‐MI group (*P* < .05) (Figure 2A). Serum miRNA‐22 levels were reduced significantly in the AMI group compared to the non‐MI group (*P* < .05) (Figure 2B). Moreover, there was a statistically significant difference in miRNA‐499 between non‐MI and AMI (*P* < .05).

### 3.3 Analysis of the sensitivity and specificity of miRNA‐499 and miRNA‐22

**FIGURE 3:**
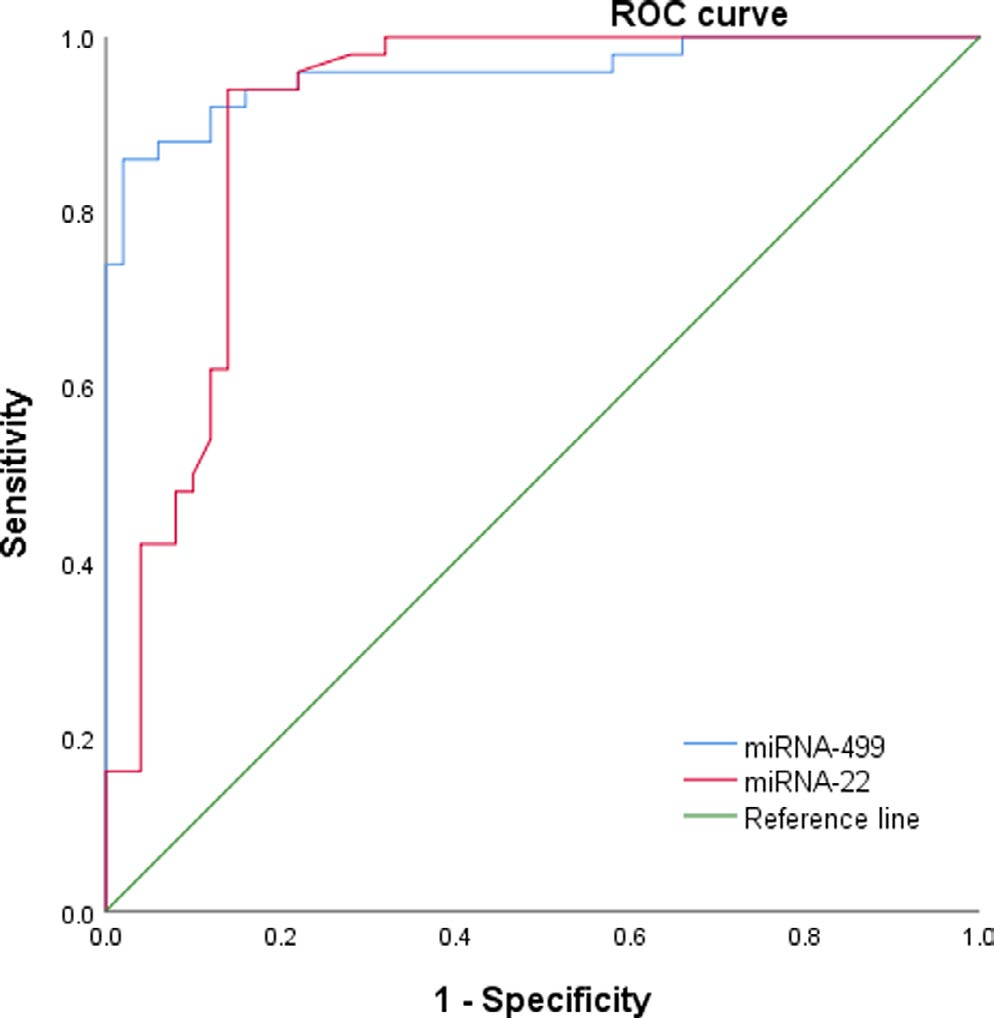
ROC curves were constructed to compare the relative concentrations of miRNA‐499 and miRNA‐22

The receiver operating characteristic (ROC) curves were plotted with (1‐specificity) as the abscissa and sensitivity as the ordinate. Furthermore, whether the miRNAs can be used as diagnostic markers for AMI can be analyzed by the ROC curve and the area under the ROC curve (AUC). Therefore, the ROC curves of non‐myocardial populations were plotted to analyze the diagnostic efficacy of miRNA‐499 and miRNA‐22 for AMI. As shown in Figure 3, the area under the ROC curve of miRNA‐499 and miRNA‐22 (AUC) was 0.959 (95% CI: 0.921‐0.997) and 0.908 (95% CI: 0.845‐0.971), respectively. When the miRNA‐499 level of AMI patients was 0.377, the sensitivity of miRNA‐499 in diagnosing AMI was 86.00% and the specificity was 98.00%. When the miRNA‐22 level was 0.946, the sensitivity of miRNA‐22 in diagnosing AMI was 86.00% and the specificity was 94.00%.

### 3.4 Logistic regression analysis

Logistic regression analysis showed that miRNA‐499 and miRNA‐22 were independent predictors of AMI (miRNA‐499: *P* = .020; miRNA‐22: *P* = .011) (Table 3). Through the logical regression analysis, a regression model was constructed to diagnose AMI from non‐MI population. The logistic regression model was as follows:

$$\text{Logit}\;\left(P=\text{AMI}\right)=0.196+5.528\times \text{miRNA}-499-2.709\times \text{miRNA}-22$$

**Table 3:** Correlation analysis between miRNAs and various indicators

Variables	miRNA‐499		miRNA‐22	
	*r*	*P*	*r*	*P*
WBC (10^9^/L)	.442	.000	−.405	.004
RBC (10^12^/L)	.256	.027	−.283	.014
Hb (g/L)	.252	.029	−.214	.065
PLT (10^9^/L)	−.112	.338	−.008	.945
TC (mmol/L)	.322	.005	−.206	.076
TG (mmol/L)	.051	.662	−.054	.643
BUN (mmol/L)	.135	.249	−.159	.173
Cr (µmol/L)	−.011	.924	−.016	.891
CK (U/L)	.525	.000	−.367	.001
CK‐MB (U/L)	.519	.000	−.377	.001
miRNA‐499	—	—	−.393	.000
miRNA‐22	−..393	.000	—	—

Abbreviations: CK, creatine kinase; CK‐MB, creatine kinase‐MB.

**FIGURE 4:**
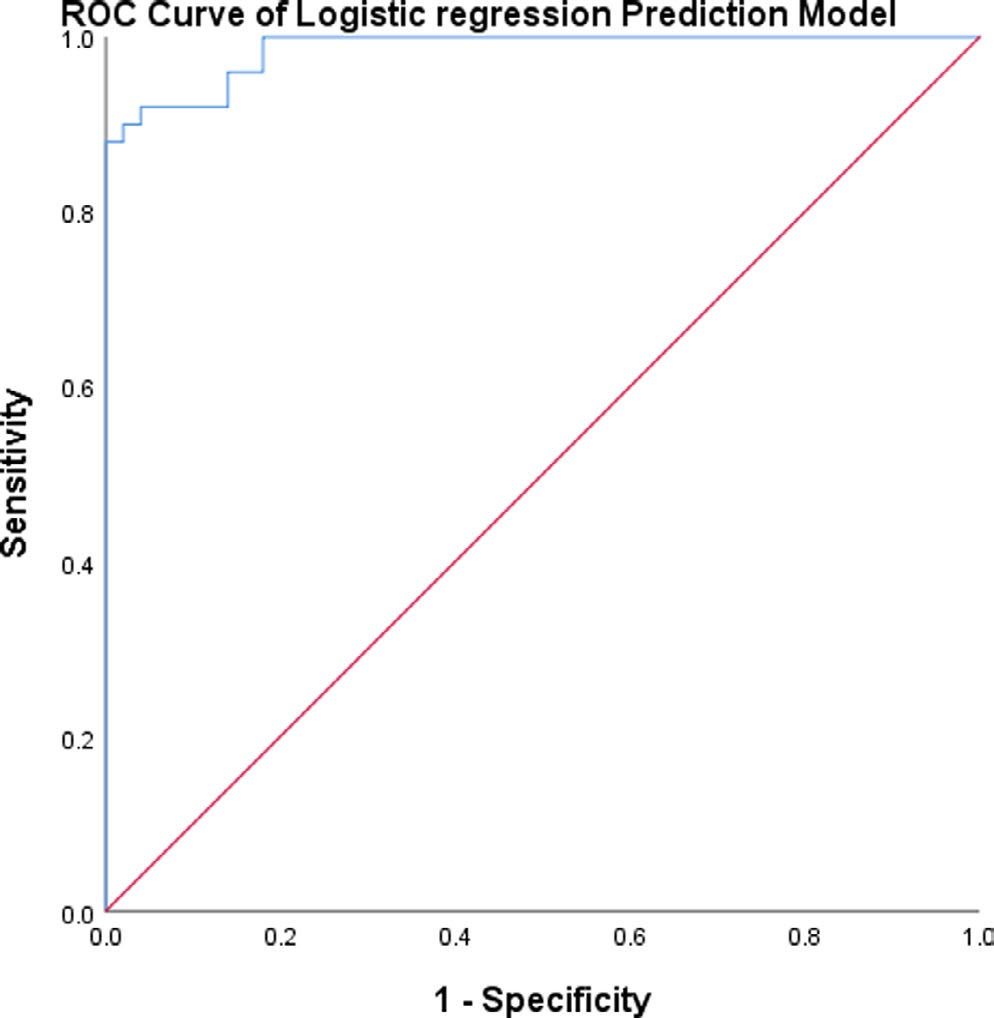
Receiver operating characteristic curve analysis with logistic regression prediction model

Results showed that the cutoff value was 0.1511 with maximizing Youden index. According to the regression model, the sensitivity and specificity for the diagnosis of AMI were 98.00% and 96.00%, respectively (Figure 4).

In order to further verify the ability of model testing AMI, 40 samples (20 AMI, 10 UAP + 10 HC) were tested by a blind method. Finally, the true‐positive rate was 90% and the true‐negative rate was 100%.

### 3.5 Correlation analysis

Spearman rank correlation analysis was used to analyze the relationship between miRNAs and various indicators in AMI and UAP patients. The results are shown in Table 4. There was a positive correlation between miRNA‐499 and white blood cell count (*r* = .442, *P* = .000), and miRNA‐22 was negatively correlated with white blood cell count (*r* = −.405, *P* = .004). In addition, the positive correlation between miRNA‐499 and total cholesterol levels was observed (*r* = .322, *P* = .005). The results indicated that miRNA‐499 exhibited a significantly positive correlation with CK (*r* = .525, *P* = .000) and CK‐MB (*r* = .519, *P* = .000), respectively (Figure 4). In contrast, miRNA‐22 levels were significantly negatively correlated with CK (*r* = −.367, *P* = .001) and CK‐MB (*r* = −.377, *P* = .001), respectively. Interestingly, there was a significant correlation between miRNA‐499 and miRNA‐22 with a correlation coefficient of *r* = −.393 (*P* = .000).

**Table 4:** Comparison in MCAE and non‐MACE groups

Variables	Non‐MACE	MACE	*P*
miRNA‐499	1.06 ± 1.33	2.73 ± 1.53	.000
miRNA‐22	0.82 ± 0.69	0.35 ± 0.28	.029
GRACE score	83.91 ± 24.97	126.27 ± 39.15	.001

### 3.6 The predictive value of miRNAs, GRACE score, and combined indicator of miRNAs and GRACE score

**FIGURE 5:**
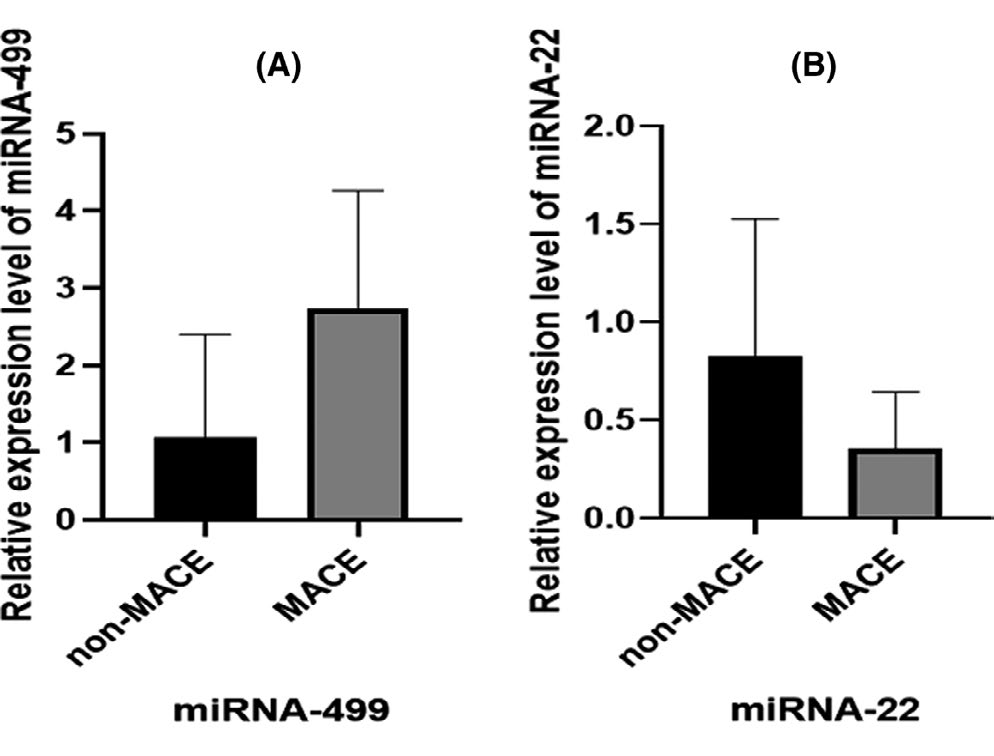
miRNA‐499 and miRNA‐22 levels in the MACE and non‐MACE groups

Data analysis was performed between the MACE group and the non‐MACE group. Compared with the non‐MACE group, the MACE group had higher levels of serum miRNA‐499 and GRACE score (*P* < .05) (Figure 5A). Conversely, miRNA‐22 levels in the MACE group were significantly lower than in the non‐MACE group (*P* < .05) (Figure 5B). As shown in Table 5, the average levels of miRNA‐499 in the MACE and non‐MACE groups were 2.73 ± 1.53 and 1.06 ± 1.33, respectively (*P* < .05). Moreover, the mean value of miRNA‐22 between the MACE group and the non‐MACE group was 0.35 ± 0.28 and 0.82 ± 0.69 which was statistically different (*P* < .05).

**Table 5:** AUC (95% CI), optimal cutoff value, and corresponding sensitivity and specificity by ROC curve analysis

Variables	AUC (95% CI)	Cutoff value	Sensitivity, %	Specificity, %
miRNA‐499	0.822 (0.728‐0.916)	1.30	100.00	69.80
miRNA‐22	0.700 (0.570‐0.830)	0.78	44.40	100.00
GRACE score	0.814 (0.703‐0.925)	94.75	91.70	63.50
miRNA‐499 + miRNA‐22	0.844 (0.749‐0.939)	—	91.70	74.60
miRNA‐499 + miRNA‐22 +GRACE score	0.929 (0.866‐0.991)	—	100.00	79.40

**FIGURE 6:**
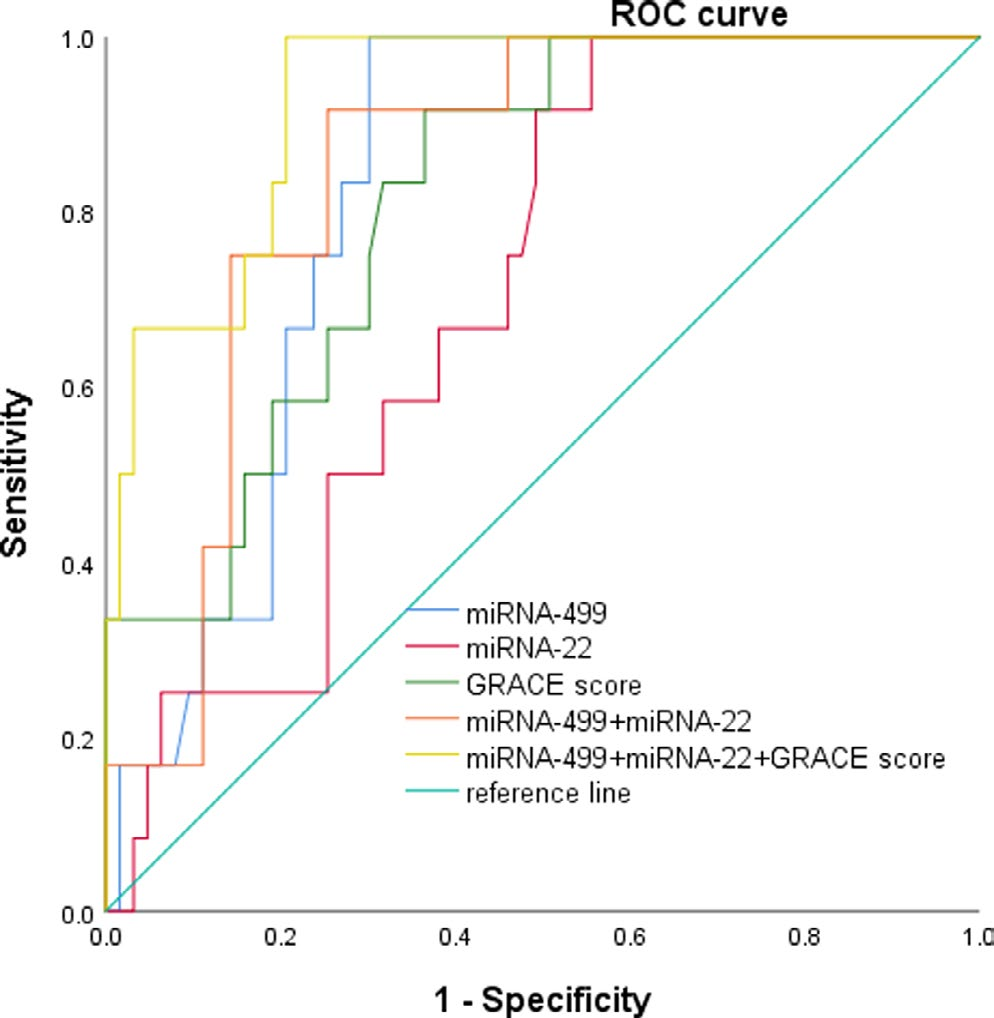
Receiver operating characteristic curve analysis with different type of predictive indicator

The ROC analysis results are shown in Table 5, and the AUC areas of miRNA‐499, miRNA‐22, and GRACE score were 0.822, 0.700, and 0.814, respectively. In addition, combining miRNA‐499 and miRNA‐22 reached the AUC of 0.844, which was higher than miRNA‐499 or miRNA‐22 alone. Furthermore, compared with the baseline GRACE scoring system, the combination of miRNA‐499, miRNA‐22, and GRACE score had a stronger discriminative ability for cardiovascular disease risk, with a sensitivity of 100% and a specificity of 79.40% (Figure 6).

## 4 DISCUSSION

Acute myocardial infarction (AMI) caused by myocardial ischemia is the leading cause of morbidity and mortality in the world.^19^ For patients with AMI, accurate diagnosis and timely treatment are of paramount importance.^25^ At present, some common indicators, such as creatine kinase‐MB (CK‐MB) and highly sensitive cardiac troponin (hs‐cnTnT), have become biomarkers for the diagnosis of AMI,^26^ but their sensitivity and specificity are still not satisfactory. Some studies have shown that circulating miRNAs are specifically expressed in tissues and participate in the pathological process of myocardial infarction.^27^, ^28^ In addition, based on the remarkable stability of miRNAs in plasma, the potential of miRNAs as markers of myocardial infarction is further illustrated.^29^, ^30^

Recently, it has been found that miRNA‐499 is one of the miRNAs encoding myosin.^31^ MiRNA‐499 is located in the intron of b‐myosin heavy chain 7B (Myh7b) gene in the human heart.^32^ Moreover, miRNA‐499a is highly expressed in the heart.^33^ Similarly, Wang et al^34^ have shown that miRNA‐499 is produced almost exclusively in the heart. Jia et al^35^ found that miRNA‐499 may be involved in myocardial injury and remodeling. In addition, miRNA‐499 was shown to be involved in cardiomyocyte differentiation.^36^ Wilson et al^37^ believe that the overexpression of miRNA‐499 will lead to the up‐regulation of cardiac myosin heavy chain gene. Also, miRNA‐499 has been shown to regulate the expression of many inflammatory cytokines, including IL‐17Rβ, IL‐23α, IL‐2R, IL‐6, IL‐2, and IL‐18R.^38^ This study found that the relative expression level of miRNA‐499 was increasing between HC, UAP, and AMI. Moreover, the sensitivity of miRNA‐499 for diagnosing AMI was 86.00%, the specificity was 98.00%, and the AUC was 0.959. The value of 0.377 further demonstrates the diagnostic value of miRNA‐499 in AMI. In addition, our results also found that miRNA‐499 exhibited a significantly positive correlation with CK (*r* = .525, *P* = .000) and CK‐MB (*r* = .519, *P* = .000), respectively. In addition, the average levels of miRNA‐499 in the MACE and non‐MACE groups were 2.73 ± 1.53 and 1.06 ± 1.33, respectively (*P* < .05). According to Figure 5A, the area under the curve (AUC) of miRNA‐499 for the MCAE was 0.822(0.728‐0.916), with an optional cutoff value of 1.30, sensitivity of 100.00%, and specificity of 69.80%.

At present, many studies have shown that miRNA‐22 plays an important role in cardiovascular diseases.^39^ Moreover, Hu et al believed that miRNA‐22 was abundant in myocardial tissue.^40^ Tu et al^41^ found that when cardiomyocytes responded to stress, the expression of miRNA‐22 in the heart increased moderately, which led to an increase in circulating miRNA‐22 in plasma. Similarly, Li et al^42^ agreed with this view. In addition, Maciejak et al^43^ found that miRNA‐22‐5p was up‐regulated in the acute phase of STEMI. In contrast, in our study, serum levels of miRNA‐22 showed a downward trend with disease progression. The level of miRNA‐22 in the UAP group was significantly lower than that in the HC group, while the AMI group was significantly lower than the UAP group. The ROC curve showed that the sensitivity of miRNA‐22 in diagnosing AMI was 86.00% and the specificity was 94.00%, further indicating that miRNA‐22 has the potential to diagnose AMI. Our results are consistent with those reported by Wang Y et al^44^ The reason for the inconsistency of miRNA‐22 changed in different literature may be related to the differences in the research object. Furthermore, the mean value of miRNA‐22 between the MACE group and the non‐MACE group was 0.35 ± 0.28 and 0.82 ± 0.69, which was statistically different (*P* < .05). Similarly, the AUC of miRNA‐22 was 0.700 (95% CI = 0.570‐0.830) with an optional cutoff value of 0.78, sensitivity of 44.40%, and specificity of 100.00% (Figure 5B). Li G et al^45^ believed that overexpressed miRNA‐22 could protect cardiomyocytes by promoting autophagy and inhibiting apoptosis. Therefore, at the time of myocardial infarction, the reduction of miRNA‐22 had the opposite effect, which in turn caused damage to the cardiomyocytes. It was noteworthy that Yang et al^46^ also believed that miRNA‐22 was inversely related to its target genes MECP2 and EVI1. It was further demonstrated that miRNA‐22 may provide a potential clinical application for the treatment of cardiovascular diseases.

There are still some limitations to our research. First, the number of samples in the current study is relatively small. Second, miRNAs change with the progression of myocardial infarction, and miRNAs expression should be detected at different times. Third, the level of miRNAs should be measured dynamically and continuously as much as possible in subsequent experiments.

## 5 CONCLUSIONS

In conclusion, miRNA‐499 and miRNA‐22 may be potential biomarkers for the diagnosis of AMI, and miRNA‐22 may be a potential therapeutic agent for coronary atherosclerosis. And with the improvement of miRNAs detection technology, more accurate, fast, and inexpensive detection methods may appear. At the same time, taking into account the limitations of the detection time limit and the number of samples, further research is needed to confirm the results.

## CONFLICT OF INTERESTS

The authors declared no potential conflicts of interest with respect to the research, authorship, and/or publication of this article.

## AUTHOR CONTRIBUTIONS

Xiaoqing Wang conceived the research, participated in the design of the research, participated in data analysis and interpretation, and helped draft the article for important intellectual content. Lu Tian participated in research design and data collection, and helped draft the article for important intellectual content. Qiyu Sun conceived the research, participated in research design, participated in data analysis and interpretation, and revised the article. Guarantor: Qiyu Sun.

## ETHICAL APPROVAL

Medical Ethics Committee of the Hospital Affiliated to Chengde Medical University (Research No. 20801A052).
